# The evolution of psychotherapy: from Freud to prescription digital therapeutics

**DOI:** 10.3389/fpsyt.2024.1477543

**Published:** 2024-10-23

**Authors:** John P. Docherty, Brett M. Colbert

**Affiliations:** ^1^ Department of Psychiatry, Weill Cornell Medical College, New York, NY, United States; ^2^ Medical Scientist Training Program, University of Miami Miller School of Medicine, Miami, FL, United States

**Keywords:** psychotherapy, digital therapeutics, software as a medical device, review, prescription digital therapeutic (PDT)

## Abstract

The evolution of psychotherapeutic treatments from Freud to digitally administered evidence-based treatments reflects a history of progressive advance. This history is characterized by identification of problems with the current state of the art, followed by solutions inspired and supported by advances in basic science and technology leading to subsequent recognition of other limitations revealed by the new advance. The common thread running through this process is (a) increasing specificity of the psychotherapeutic interventions, (b) increasing evidence of efficacy and safety, (c) increasing integrity and reliability in the delivery of the intervention, (d) increased equality of access, and (e) recognition of the need for regulation to provide protection for the public from unsafe or ineffective products. This evolution of psychotherapeutic treatments, not surprisingly, has been foreshadowed by the precursor history of the evolution of pharmacologic treatment. Although intellectual history is lumpy and does not sort itself into discrete and coherent epochs, such sorting is a useful heuristic for describing the advance of medicine and the therapeutic enterprise. This paper will discuss six successive epochs of psychotherapy. For each it will discuss the problem of the preceding era it sought to solve, the advance it brought to the field, the emerging science and technology that supported that advance, and the precursor development in pharmacological treatments that foreshadowed that epoch of psychotherapy. Finally, it will conclude with some observations about the proximate future.

## Introduction

## Phase 1: Freud (1890-1950)

**The Problem:** Psychotherapy before Freud was a mix of moral, somatic, and early psychological approaches, heavily influenced by religious and spiritual beliefs. The interventions lacked an explanatory model of mental illness that could explain the development and manifestation of psychological disorders. Treatments were largely symptom-focused and atheoretical. For example, an enlightened approach was Moral Therapy practiced by Philippe Pinel in France and by the Tukes in England. The basic principles, which are still helpful, included adequate living conditions, respectful attitude, and clarity of communication and organization. This approach was not, however, grounded in a systematic approach to understanding, remediating, or curing mental illness (1, 2).

**The Advance:** Freud provided an organized theory of mental illness and a systematic approach including strategies and techniques for the practice of psychotherapy. He proposed that early experiences, unconscious processes, and inner conflicts contribute to mental health disorders. In essence, he provided a rationale for why a “talking cure” therapy could be effective, when it was needed, and how to systematically apply that therapy. He introduced the first major systematic form of psychotherapy and, in so doing, revolutionized the field (3, 4).

**Science and Technology:** Sigmund Freud’s development of psychoanalysis wasn’t directly preceded by a specific advance in basic science, but it was influenced by various scientific and intellectual developments of the 19th century. Some of the key influences included advances in neurology, particularly the work of Jean-Martin Charcot and Josef Breuer. Charcot’s studies on hysteria and hypnosis suggested that mental processes could affect physical symptoms. Breuer’s work with the “talking cure” (cathartic method) with the patient Anna O. (Bertha Pappenheim) laid the groundwork for Freud’s ideas about the therapeutic potential of talking (5, 6).

The philosophical works of Arthur Schopenhauer and Friedrich Nietzsche, as well as literary explorations of the human psyche by authors like Fyodor Dostoevsky, also influenced Freud. These philosophers and novelists explored themes of irrationality, the unconscious, and the conflict between drives and societal norms. While there wasn’t a single breakthrough in basic science that directly led to Freud’s work, these various streams of thought and research contributed an environment for the development of psychoanalysis (7–9).

**The Pharmaceutical Parallel Precursor:** Interestingly the evolution of pharmacology has regularly preceded that of psychotherapy by several decades. For example, the 18th and 19th centuries represent a pivotal era in pharmacology, a transition from traditional herbal remedies to scientifically based medicine. Germany was the center of this advance. This advance built on the prior half-century’s progress in organic chemistry and the ability to isolate and characterize therapeutically effective compounds, such as the extraction of morphine from opium by Friedrich Sertürner in 1804. In the latter part of the century, paralleling the contribution of Freud to psychotherapy, Rudolf Buchheim established the first pharmacological institute in 1847 in Germany, focusing on the systematic study of the effects of drugs. Oswald Schmiedeberg, expanded this work, emphasizing the importance of understanding drug actions and effects (10, 11).

## Phase 2: Science-derived psychotherapies (1950-1970)

**Problem**: Although Freud and his elaborators such as Carl Jung, Alfred Adler, and others enhanced the application of Freud’s theory of psychotherapy, the elaborations were primarily based on clinical observation and theoretical speculation and lacked a broader grounding in science.

**Advance**: Robust development in several areas of psychological science led to the development of a set of therapies informed by clinical practice and observation but derived from more fundamental psychological science. Psychotherapeutic interventions such as Behavioral Therapy (Eysenck, Wolpe) emerged, focusing on observable behaviors rather than internal mental states. B.F. Skinner, Joseph Wolpe, and Hans Eysenck were major contributors to developing these methods. Other innovative therapies such as Cognitive Therapies (Beck, Lazarus, Ellis), and Family and Social System Therapies (Bateson, Minuchin) were also developed during this time.

**Science and Technology**: Important developments of this period included the work of B.F. Skinner and others in behavioral psychology and learning theory, the development of cognitive psychology and information processing, including the work of George Miller (“the Magical Number Seven, Plus or Minus Two”) and Ullrich Neisser, and the development of systems theory represented by Von Bertalanffy’s foundational work and Albert Bandura’s work on social learning and self-efficacy (12–23).

In addition, the 1950s and 1960s saw a revolution in the treatment of mental disorders through the discovery and development of psychoactive drugs. This period, often referred to as the “psychopharmacological revolution,” had a profound impact on the field of psychiatry and psychotherapy. Major advances in psychopharmacology, including identification of the antipsychotic effect of chlorpromazine and the antidepressant effect of imipramine, supported the development of a biopsychosocial model of psychiatric illness. The biopsychosocial model promoted a dynamic and interactional approach which recognized the importance of social stress and adverse life-experience for precipitating states of mental distress (21). This led to therapies such as the Interpersonal Psychotherapy of Klerman and Weissman and Pleasant Events therapy of Lewinsohn (22–25).

**The Pharmaceutical Precursor Parallel:** The developments of chemistry and pharmaceutical science of the late 19th and early 20th centuries lay the basis for the development of synthetic drugs. This marked a departure from drugs derived purely from natural sources and opened the door to the development of whole new classes of medications. The late 19th and early 20th centuries also saw significant advancements in understanding how drugs interact with the body (pharmacodynamics) and how the body affects drugs (pharmacokinetics). This included studying receptor theory and drug metabolism, which are fundamental to modern pharmacology. These science-based advances in pharmacology in the second half of the 19^th^ century presaged the science-based developments in psychotherapy that were to occur for psychotherapy in the mid- 20^th^ century (9, 26, 27).

## Phase 3: Evidence-based psychotherapy - part one: initial empirical studies (1970-1980)

**Problem**: Psychotherapy lacked a robust valid body of knowledge demonstrating how psychotherapy worked and how well it worked. There was an increasing recognition of the need for empirical evidence by clinicians, researchers, and policymakers. Hans Eysenck’s 1952 paper challenging the effectiveness of psychotherapy initiated more rigorous research methods in the field. It highlighted the need for more well-designed, controlled trials to credibly assess the efficacy of psychotherapy (12).

**Advances**: The recognition of this knowledge gap led to an increasing number of studies on the clinical outcomes of psychotherapeutic interventions. Randomized Controlled Trials (RCTs) became the gold standard for evaluating psychotherapy effectiveness. The increasing use of clinical trials to test the efficacy of psychotherapy in the 1960s and 1970s marked a significant shift in the scientific study and validation of psychological treatments. Although still suffering from methodological flaws and limitations, clinical trials became the norm. By the late 1970s, the added development of meta-analytic techniques allowed researchers to aggregate results from multiple studies, providing more robust evidence indicating the effectiveness of psychotherapy across various conditions (28, 29).

In synchrony with this increased attention to outcomes research, there was an increasing focus on process research—empirical evaluations of the putative mechanisms through which psychotherapy exerts its effects. The 1960s and 1970s saw the emergence of new methodologies and theoretical frameworks for conducting process research. Carl Rogers, the developer of client-centered therapy, emphasized the therapeutic relationship and the role of empathy and unconditional positive regard in facilitating client change, spurring studies exploring these therapeutic processes. During this time, Systematic methods for observing and coding therapy sessions emerged. For example, session rating scales and behavioral coding systems were used to analyze therapist and client behaviors. Advances in technology, which allowed for the recording of therapy sessions, enabled researchers to study non-verbal behaviors and other aspects of the therapeutic process. One of the most important areas of process research and a major contributor across psychotherapeutic modalities was the therapeutic alliance. Studies began to investigate how the therapeutic alliance—the collaborative bond between therapist and client—influenced therapy outcomes. Additionally, the development of reliable and valid measurement tools, such as the Working Alliance Inventory (Horvath & Greenberg, 1989), allowed for more systematic and empirical investigation of process variables. Jerome Frank was a seminal contributor to this area of work, making a lasting contribution to the field by identifying the common factors of psychotherapies effectiveness including the alliance, evoking of emotion, change of meaning and reversal of demoralization (30–35).

**Science and Technology**: The development and refinement of psychological assessment instruments supported more reliable diagnosis and measurement of treatment outcomes. Developments in the methodology of clinical trials, such as advances in statistical methods for RCTs, increased the number of high-quality psychotherapy efficacy studies. Key advances included refinements in randomization techniques, such as random number tables, computerized randomization, and stratified randomization to control for confounding variables. Other advances were improved double-blind procedures, methods of power analysis to determine appropriate study sample size, multivariate analysis, intention-to-treat (ITT) analysis as well as ethical advances including informed consent and institutional review boards. All in all, it was a period with a transformational shift in attention to the importance of clinical evidence (36, 37).

**Pharmacology Precursor Parallels**: As early as the mid to late 19^th^ century there was attention to methods for testing drugs, including the use of animal models to study the physiological and toxicological effects of substances. These methods laid the foundation for clinical pharmacology and toxicology. Randomized controlled trials (RCTs) became a standard practice for testing the efficacy and safety of medications in the mid-20th century. The pivotal shift towards RCTs as the gold standard began in the late 1940s and early 1950s. One of the earliest and most influential examples of an RCT was the 1948 study on the antibiotic streptomycin for the treatment of tuberculosis. This trial is considered a landmark because it used random allocation, controls, and blinding to minimize biases. The adoption of RCTs became more widespread in the following decades, particularly with the establishment of regulatory agencies like the U.S. Food and Drug Administration (FDA) and the development of ethical guidelines and standards for conducting clinical research. By the 1960s and 1970s, RCTs had become routine and were considered the standard method for evaluating the efficacy and safety of new medications before approval for general use (38, 39).

## Phase 4: Evidence-based psychotherapy - part two: standardization (1980-2005)

**Problem**: Following the advances of the preceding epoch, two problems came into focus. The first was the need for greater standardization of specific therapies The second was a progressive realization of certain limitations with standardization.

*Need for Standardization:* In the studies of the prior period, it was generally not possible to know what therapists had actually done in delivering therapeutic treatment. For example, an excellent and widely-cited review of comparative trials published in 1975 did not include specification and standardization of the therapy as a critical aspect of the study (40). This lack of clarity and specification of the treatment (the experimental variable) made it impossible to compare therapies. In contrast, the experimental variable in a drug trial, the drug, can be precisely described. In comparative drug trials, the groups of subjects, the treatments, and which groups are getting which treatments are clearly known. Psychotherapy trials lacked a way of ascertaining or ensuring that this state-of-affairs was similarly true. The field lacked a method to ensure therapeutic integrity and reproducibility, that is, certainty that therapies could be consistently applied across different settings and by different therapists (41–44).

*Limitations of the Standardization of Psychotherapies*: Successfully standardized psychotherapies, such as the initial form of cognitive behavioral therapy (CBT), although recognized as effective, also were perceived to have salient limitations affecting patient acceptance and broader effectiveness. The so-called “technological” model of these standardized psychotherapies emphasized techniques and protocols in a manner similar to medical or technological procedures. The strict focus on standardization was progressively seen to be at the expense of other considerations essential to realizing the full potential of psychotherapy, such as the centrality of the therapeutic relationships, the patient’s context and its impact, and the patient’s lived experience (41–44).

**Advance**: The first advance of this epoch was the development of a set of techniques to assist in the standardization and reproducibility of a specific therapy. The 1970s saw the introduction of treatment manuals, which provided detailed guidelines for therapists to follow, making it possible to replicate and test therapies in controlled studies. These manuals specified the strategies and techniques that constituted the therapy (what was prescribed) and the strategies and techniques that were not part of the therapy (what was proscribed). Manuals, however helpful, were not sufficient to achieve adequate standardization. This gap led to the development of additional methods including (a) treatment protocols that provided specific, detailed instructions for therapists on how to deliver a particular therapy, (b) training and certification programs including reliable assessments of therapist competency, (c) fidelity measures to assess if the therapist is adhering to the manual and treatment protocol and that the therapy is being delivered as intended which could be used on a session-by-session basis, (d) peer review meetings to prevent and rectify therapist drift, (e) expert review and rating of competency of each session with supervisory intervention if competency fell below a predetermined level (45).

In the early 1980s, The Psychosocial Treatment Research Branch of the National Institute of Mental Health (NIMH) launched the Treatment of Depression Collaborative Research Program which employed the methods noted above in a multisite clinical trial comparing Cognitive Behavioral Therapy, Interpersonal Psychotherapy, Imipramine and Placebo in the treatment of Major Depressive Disorder. The success of this trial helped firmly establish these standardization methods as necessary elements of a psychotherapy clinical trial. The NIMH and National Institute on Drug Abuse (NIDA) subsequently facilitated the use of standardization methodology by developing funding mechanisms for novel treatment manual development, therapist certification training, fidelity scale development, and pilot testing. These methodological advances led to a greater emphasis on empirically supported treatments and contributed to the development of numerous evidence-based psychotherapies. The American Psychological Association (APA) began to emphasize the identification of ESTs, therapies that had been proven effective through controlled research. Lists of ESTs were compiled to guide clinical practice and research. Today, the current American Psychological Association Society of Clinical Psychology (Division 12) website lists 89 therapies with varying strength of evidence that have been evaluated using at least one of two sets of criteria (46–59).

The second advance was the robust development of the so-called third generation psychotherapies with demonstrated efficacy. These new therapies included Acceptance and Commitment Therapy, Compassion-Focused Therapy, Mindfulness-Based Cognitive Therapy, Schema Therapy, Dialectical Behavior Therapy and Meta-Cognitive Therapy (53–59). These therapies were designed to address the omissions and limitations of the psychotherapies that adhered closely to a technological model, including an overemphasis on cognitive restructuring, inadequate attention to emotion and context, difficulties with client engagement, and cultural rigidity. These newer approaches sought to integrate mindfulness, acceptance, values, and a more holistic understanding of human experience into therapeutic practice, addressing the gaps left by traditional CBT.

**Science and Technology**: The development of standardization methodologies for psychotherapy occurred in an environment and zeitgeist of standardization which included major initiatives such at the American Psychiatric Association’s Diagnostic and Statistical Manual III (DSM-III), whose intent was to standardize the diagnostic criteria for all psychiatric disorders. The publication of the DSM-III in 1980 facilitated more consistent and reproducible identification of mental health disorders. It provided a common language for diagnosing and treating psychological conditions, which was crucial for developing standardized treatment protocols. Further, structured clinical interviews were developed to ensure that, in addition to criteria variance, information variance was also reduced. At the same time, a similar development was taking place for disorder severity rating scales (e.g. the Hamilton Depression Rating Scale) (60).

As often happens this process of standardization was simultaneously happening in other areas of science and technology. In fact, this process was widespread. For example, during the 1980s, in information technology (IT), software engineering formed as a discipline, leading to the establishment of programming standards, protocols, and best practices. The standardization of networking protocols, most notably Transmission Control Protocol/Internet Protocol (TCP/IP), facilitated the development of the internet and improved interoperability between different computer systems and networks. In biology, standardized laboratory techniques, such as polymerase chain reaction (PCR), became essential tools in biotechnology and molecular biology. In environmental science, the standardization of environmental monitoring techniques, such as the use of consistent protocols for air and water quality testing, became increasingly important. Similarly, the introduction of standardized food safety regulations, such as Hazard Analysis and Critical Control Points (HACCP), aimed to ensure the safety and quality of food products (61–65).

**Pharmacology Precursor Parallel**: The standardization parallel in pharmacology was the development and implementation of Good Manufacturing Practices (GMP), Good Laboratory Practices (GLP), and Good Clinical Practices (GCP). These standards were established to ensure the safety, quality, and efficacy of pharmaceutical products and to standardize the processes involved in their development, testing, and manufacturing. The modern concept of GMP began to take shape in the late 1960s and 1970s, with the FDA publishing the first comprehensive GMP regulations in 1963. The World Health Organization (WHO) and other international bodies adopted these principles. GLP standards, which followed in the 1970s, were designed to ensure the quality and integrity of non-clinical laboratory studies. These practices cover the organization, process, and conditions under which laboratory studies are planned, performed, monitored, recorded, and reported (66–69).

## Phase 5: Digital psychotherapy (2005-2015)

**The Problem**: The advances in psychotherapy standardization supported the formation of a body of evidence-based psychotherapies. Unfortunately, two major problems related to the effective delivery of these highly structured treatments remained.

First, delivery of these evidence-based therapies in current practice relied on unobserved individual practitioners presumably adhering to the specifications of the therapy that is purportedly being delivered—a challenging proposition. Akin to rater drift, there is the phenomenon of therapist drift, which is the tendency of human beings, without external constraints and support, to drift to idiosyncratic ways of doing things. As a result, despite having an evidence base of efficacious standardized therapies, the absence of the considerable support structure of a well-constructed clinical trial meant unknown levels of variance in the effective delivery of these therapies (70, 71).

Second, even if individual practitioners were delivering the evidence-based psychotherapy as it was designed and tested, there was an additional problem with this method of delivery—it is subject to an inherent supply-demand mismatch. It is not possible to have a sufficient number of well-trained evidence-based psychotherapists to deliver the treatment to everyone in need. For example, as of December 2023, over half (169 million) of the U.S. population lives in a Mental Health Professional Shortage Area (MHPSA) (72–74).

**The Advance**: Almost as a rule, ethical advance and technological advance walk hand-in-hand. Put another way, ethical imperatives often wait on technological advances. In the 1980s, there was a recognition of both problems noted above. However, the 1980s had yet to experience the significant current advances in telecommunications and the digital delivery of audio, visual, and written information. There was no internet and no iPhone. The Sony Walkman was too limited to deliver a complex psychotherapy. The early 21^st^ century saw a transformation of daily life due to the internet and smartphones. With specific regard to psychotherapy, technological advances—first internet-based treatment, then treatment delivered by a smartphone—have facilitated the maintenance of the integrity a of evidence-based psychotherapies and their democratized distribution. Programs like the “FearFighter” for anxiety disorders and “Beating the Blues” for depression emerged, offering structured CBT via computer platforms. It is difficult to determine the first digital psychotherapy app, but two are contenders. In 2010, My Compass was released by the Black Dog Institute in Australia and Headspace was founded in London. However, estimates suggest there are now 10,000 to 20,000 mental health apps publicly available. These apps cover a wide range of functions, including mindfulness and meditation, therapy support, mood tracking, and more. This sudden plethora of products in mental health and other areas of medicine has led to a broad recognition of the need for quality control. That understanding has, subsequently, led to prescription digital therapeutics (which will be discussed in the next section) through the FDA’s recognition of the need to regulate this rapidly expanding arena of novel therapeutic devices (75–79).

**Science and Technology:** The ability to produce psychotherapies in a digital mode was made possible, as noted above, by the extraordinary advances in information and communications technology. These include the internet, smartphone, and cloud computing which provided the infrastructure to support the storage and ubiquitous access of large amounts of data, enabling scalable digital health platform. Relevant also was the development of cybersecurity and compliance standards, such as HIPAA, to protect sensitive personal health information (80, 81).

**Pharmacology Precursor Parallels**: Mass Production and Standardization. The Industrial Revolution enabled the large-scale production of drugs, which was a major shift from the artisanal preparation of medicines. This period also saw the introduction of standards for drug purity and dosage, which were crucial for ensuring safety and efficacy. The story of Bayer is particularly interesting and illustrative. In the late 1800s, Germany was at the forefront of the field of medicinal chemistry. Germany was also a leader in the development of pharmacopoeias, which set standards for drug quality and consistency. At the time, these medications were formulated in each doctor’s office (much like our individual practitioner delivery of evidence-based psychotherapy). Bayer was a manufacturer of synthetic dyes used primarily for carpets. Friedrich Bayer recognized that his company’s more fundamental expertise was in the mass production of standardized well-characterized chemical compounds. He realized that this expertise could reduce the artisanal variability of individual compounding, while at the same time increase the distribution and decrease the cost of medications. The company’s first pharmaceutical product was acetylsalicylic acid, which was successfully synthesized in a stable form in 1897 and which Bayer introduced under the brand name “Aspirin” (82).

## Phase 6: Prescription digital therapeutics (2015-present)

**The Problem:** As noted above, the demand for mental health care and the capability of mass production resulted in an estimated 10,000 to 20,000 apps without required evaluations and assurances of quality, effectiveness, and safety. Most mental health apps are not based on evidence-based therapeutic protocols, which means their effectiveness is fundamentally questionable. A review by the Organization for the Review of Care and Health Apps (ORCHA) found that only about 29.6% of mental health apps meet quality thresholds. The percentage drops even lower for specific conditions such as bipolar disorder (9%) and OCD (5%). This indicates a high potential for users to engage with apps that may not provide reliable or effective support, and in some cases, could exacerbate their conditions. A review of over 100 apps proposing to offer cognitive-behavioral therapy (CBT) or behavioral activation (BA) found that only 10% contained features consistent with CBT and BA. Further, there are significant patient privacy concerns. A study by the software developer Mozilla found that 28 out of 32 popular mental health apps had significant data privacy issues, often lacking robust security measures and sometimes selling user data to third parties. Lacking proper regulation, there’s no guarantee that the therapeutic content provided by these apps is based on evidence-based practices or that patient privacy is properly protected (83–85).

**The Advance:** This is work still in progress. The rapid unregulated growth of digital mental health apps led to the field being characterized as a “Wild West.” In fact, this field did and still does resemble the growth of the American Wild West. The West grew rapidly and without organized law and order. The initial attempts to establish law and order involved citizen and vigilante groups enforcing their own versions of law and order. This bears similarity to the contemporary emergence of self-appointed or semi-official guardians of mental app quality. A second phase of the American West established officially elected local law enforcement roles, such as sheriffs and marshals who operated with varying degrees of authority and effectiveness. This phase bears similarity to some professional organizations’ efforts to evaluate the quality of available apps. The problem with citizen groups and local law enforcement, however, is the vulnerability to bias, lack of transparency, consistency, and sustainability. The next phase of law enforcement sought to rectify these limitations with the development of State and Federal entities with greater resources, accountability and enforcement capability, like the U.S. Marshals Service and the Federal Bureau of Investigation. In a similar way, with the recognition of the growing risk inherent in providing devices to effect therapeutic gain in an unregulated field, the FDA initiated a review and regulatory process (86–89).

The FDA recognized that standalone software could function as a medical device and placed Digital Psychiatry Applications in its category of Software as a Medical Device (SaMD). SaMD refers to software intended to be used for medical purposes without being part of a hardware medical device. In 2009, the FDA published guidelines on software validation focusing on ensuring that software used as or in medical devices met certain quality standards. Following are subsequent key milestones in the FDA’s approach to the regulation of SaMD (90, 91):

2013: The International Medical Device Regulators Forum (IMDRF), which includes the FDA, released a definition of SaMD. This definition emphasized that SaMD includes software intended to be used for medical purposes without being part of a hardware medical device.

2014: FDA Guidance on Mobile Medical Applications: Recognizing the proliferation of mobile apps with medical functions, the FDA issued guidance on which types of mobile apps would be considered medical devices. This was a crucial step in clarifying the scope of SaMD regulation. It clarified that any product intended to treat, cure, prevent, mitigate, diagnose disease is subject to this regulation.

2017: Digital Health Innovation Action Plan: The FDA announced a comprehensive approach to fostering digital health technologies, including SaMD. This plan included the introduction of the Pre-Certification (Pre-Cert) Pilot Program, aimed at streamlining the review process for digital health products.

2019: FDA’s Working Model for SaMD Regulation: The FDA published a working model for regulating SaMD, focusing on a “total product lifecycle” approach. This model emphasizes continuous oversight and monitoring, recognizing the iterative nature of software development.

2021: Good Machine Learning Practice (GMLP): The FDA, along with other international regulatory bodies, provided foundational principles for Good Machine Learning Practice, emphasizing the importance of transparency, performance, and risk management in AI/ML-based SaMD.

2024: Prescription Drug Use Related Software (PDURS): The FDA issued guidance for the development of software applications that directly support the use of prescription pharmacotherapies. This is opening the door to the development of drug-SaMD combinations.

Only a handful of digital health apps for mental health conditions have been approved or cleared by the FDA. In December 2018, the first app cleared through the standard approval process was for reSET-O, an app designed for the treatment of Opioid Use Disorder. Most recently, on March 20, 2024 Rejoyn was the first digital mental health app cleared for use in patients with Major Depressive Disorder (92, 93).

**Science and Technology:** The development of FDA regulation for Software as a Medical Device (SaMD) has been influenced by advances in IT science and technology. Several key areas of science and technology have supported this regulatory evolution: (a) The maturation of software engineering practices, including methodologies for software development, verification, and validation, has provided a foundation for ensuring the safety and effectiveness of SaMD. This includes techniques for risk assessment and the implementation of robust quality management systems. (b) The development of standards for data exchange and interoperability, such as HL7 and FHIR, has facilitated the integration of SaMD with other healthcare systems, enhancing their utility and scope. (c) Advances in wearable technology and sensor devices have led to new opportunities for continuous monitoring and real-time data collection, which are critical for the development of certain types of SaMD. (d) The use of real-world data (RWD) to generate real-world evidence has become increasingly important for the evaluation of SaMD. This includes evidence from clinical use, patient outcomes, and post-market surveillance. (e) The FDA and other regulatory bodies have focused on developing regulatory science to assess the safety, efficacy, and quality of SaMD. This includes creating frameworks for clinical evaluation, risk management, and benefit-risk assessment specific to software products (94–97).

**Pharmacology Precursor Parallel:** The regulation of drug safety and efficacy is a cornerstone of modern healthcare, developed through a response to a history of learning from past tragedies. Its purpose is to ensure that medications are safe, effective, and unadulterated. This hard-won knowledge serves as a requisite guide for the further development and commercialization of digital mental health therapeutics. Looking back, the development of drug regulation in the United States was driven by several key tragic events which highlighted the need for federal oversight:

1. The widespread sale of patent medicines, in the late 1800s, many of which made false claims and contained harmful ingredients. The Pure Food and Drug Act of 1906 aimed to prevent the manufacture, sale, and transportation of adulterated or misbranded foods and drugs.
2. The next major legislation was the Food, Drug, and Cosmetic Act of 1938. A liquid preparation of sulfanilamide was used to treat infections. To make this preparation palatable it had been mixed with glycerol. However, glycerol was more expensive than diethylene glycol (also known as antifreeze) so to save money a substitution was made of diethylene glycol for the glycerol, resulting in the death of over 100 people, many of them children. This act required manufacturers to provide scientific proof of a drug’s safety before it could be marketed. It also gave the FDA authority to oversee the safety of food, drugs, and cosmetics and established the New Drug Application (NDA) process.
3. Stimulated by the widespread Thalidomide birth defect tragedy, the third major legislation, the 1962 Kefauver-Harris Amendments to the 1938 law were enacted. In addition to the requirement to demonstrate safety, it now required proof of efficacy before a drug could be marketed. It also established more rigorous clinical trial procedures and informed consent requirements.
4. Learning from the past and not waiting for tragedy, regulations have been enacted to proactively address other unmet and emerging needs, such as the Good Manufacturing Practices (GMP), Good Clinical Practice (GCP), International Conference on Harmonization Guidelines for quality, safety, efficacy and other miscellaneous areas such as common terminology.

This history, hopefully, has provided the framework that can protect and ensure the integrity of evidence-based digital applications for psychiatric disorders and prevent the unfortunate exposure of the public to adulterated, ineffective, or harmful digital products (98).

## Conclusions - the digital future

We have come a long way over the last 50 years in refining, improving, and ensuring the safe and effective delivery of psychotherapy. The near future will not be without struggle and resistance but offers the possibility of exciting progress. These are some of the areas that will mark this future:

Payers and Health Equity – Initial payer resistance is expectable and appropriate. Payers cannot be expected to pay for up to 20,000 or more inadequately tested and unregulated apps nor to vet them all. The FDA’s development of the SaMD regulations and the recent landmark approvals (e.g., Rejoyn for Major Depression) represent a categorical change. This digitization allowing for the cost-effective delivery of treatment to many, which was previously restricted to the few, is a major advance in health equity. There is little basis now to resist payment for prescription digital therapeutics. Issues to now be determined are appropriate pricing and distribution models.

“Wild West” – There will not be an end to the “Wild West” of medical therapies. For example, we still have a robust industry in untested nutraceuticals. However, as the availability, utility, and experiential knowledge of prescription digital therapeutics grows, as professional and public knowledge of the difference between regulated and unregulated products increases, and as regulatory enforcement ramps up for false claims, we can expect activity to focus on creating more and better prescription digital therapeutics.

Neuroscience – There is shift occurring in the neuroscience explanatory paradigm for psychiatric disorders and their treatment. It is a shift from neurochemistry to neurocircuitry. Central to this shift is the concept of brain “plasticity,” that is, the capacity of the brain to form and unform synaptic connections, throughout life, in an activity dependent fashion. This model is, for example, fundamental to the emerging field of cognitive training. Advances based on this model and associated brain activity visualization methodologies, will bring new non-pharmacological and non-invasive therapeutics administered through digital devices. The newly cleared Rejoyn is a current example. This innovative application combines both a more traditional psychotherapy (CBT) with cognitive training (Emotional Face-Matching Task). We can expect more combinations of drug and digital intervention. Some of these will entail altering the state of the brain, perhaps with a psychedelic, to make it more amenable to psychotherapeutic or CT intervention or a drug that decreases craving for patients with addictive disorders (99–101).

The Innovation and Discovery Process for Novel Digital Therapeutics – The standardization process developed during the 1980s was a seminal contribution to the field. It made possible the development of evidence-based psychotherapies that eventuated in the over 80 therapies the American Psychological Association has qualified for that designation. However, that process of standardization—manuals, training programs and certification testing, fidelity and integrity scales, competency scales, associated rater training—is cumbersome and costly and, although a vast improvement, lacks ultimate exactitude. With digital therapeutics, it is now not needed. If a therapy is developed *de novo* in a digital form, it is inherently standardized. It is now possible to do just that.

Wearables, Real World Evidence, Data Management, AI and Personalization – Each of these five are separate areas of advance, but we can expect that they will be harmonized to achieve the goal of personalized treatment. The use of wearables is ubiquitous, and the variety of functions now made amenable to remote monitoring is extensive and growing. This information combined with ecological momentary assessment provide a rich source of data to facilitate personalization. Further the connected nature of digital applications allows for the continuous collection of data from large populations of users. Advances in data management and analysis make it feasible to store and analyze these data. AI and machine learning provide an additional resource to predict and direct a personalized intervention.

In addition to these five areas, there will be others. Hopefully, the sum of this activity will be decreased suffering from mental illness worldwide.

## Data Availability

The original contributions presented in the study are included in the article/supplementary material. Further inquiries can be directed to the corresponding author/s.

## References

[1] Scull A. The Most Solitary of Afflictions: Madness and Society in Britain, 1700-1900. New Haven, Connecticut: Yale University Press (1993).

[2] Freud S. Introductory Lectures on Psycho-Analysis, Standard Edition of the Complete Psychological Works of Sigmund Freud. Richmond, Surrey: Hogarth Press (1917).

[3] Gay P. Freud: A Life for Our Time, W.W. New York City, New York: Norton & Company (1988).

[4] Ellenberger HF. Ellenberger's comprehensive history discusses the early use of hypnosis and the work of figures like Franz Mesmer and Jean-Martin Charcot, The Discovery of the Unconscious. In: The History and Evolution of Dynamic Psychiatry. New York City, New York: Basic Books (1970).

[5] Goetz CG. Bonduelle, Michel, and Gelfand, Toby, Charcot. In: Constructing Neurology. Walton Street, Oxford: Oxford University Press (1995).

[6] Safranski R. Schopenhauer and the Wild Years of Philosophy. Cambridge, Massachusetts: Harvard University Press (1991).

[7] Yalom ID. When Nietzsche Wept: A Novel of Obsession. New York City, New York: Harper Perennial (1993).

[8] Frank J. Dostoevsky: A Writer in His Time. Princeton, New Jersey: Princeton University Press (2009).

[9] Sneader W. Drug Discovery: A History. Hoboken, New Jersey: Wiley.Quirke, V (2005).

[10] Schmiedeberg O. 1838-1921: The architect of modern pharmacology. Br Med J. (2024) 332:790–1.

[11] Skinner BF. Science and Human Behavior. London, UK: Macmillan (1953).

[12] Eysenck HJ. The effects of psychotherapy: an evaluation. J Consulting Psychol. (1952) 16:319–24. doi: 10.1037/h0063633 13000035

[13] Wolpe J. Psychotherapy by Reciprocal Inhibition. Redwood City, CA: Stanford University Press (1958).

[14] Miller GA. The magical number seven, plus or minus two: some limits on our capacity for processing information. Psychol Rev. (1956) 63:81–97. doi: 10.1037/h0043158 13310704

[15] Neisser U. Cognitive psychology. Appleton-Century-Crofts. (1967).

[16] Beck AT. Cognitive Therapy and the Emotional Disorders. Madison, CT: International Universities Press (1976).

[17] Lazarus AA. Behavior Therapy and Beyond. New York City, New York: McGraw-Hill (1971).

[18] Ellis A. Reason and Emotion in Psychotherapy. Fort Lee, New Jersey: Lyle Stuart (1962).

[19] Bateson G. Steps to an Ecology of Mind. Chicago, IL: University of Chicago Press (1972).

[20] Minuchin S. Families and Family Therapy. Cambridge, MA: Harvard University Press (1974).

[21] Healy D. The Creation of Psychopharmacology. Cambridge, MA: Harvard University Press (2002). 10.1177/0957154X04045686 15272483

[22] Bandura A Walters RH. Social learning theory. Englewood Cliffs, NJ: Prentice hall (1977).

[23] Von Bertalanffy L. General System Theory: Foundations, Development, Applications. In: Braziller G, editor. Ervin Laszlo, The Systems View of the World: A Holistic Vision for Our Time. Hampton Press, Cresskill, NJ (1996).

[24] Klerman GL Weissman MM. Interpersonal Psychotherapy of Depression. New York City, New York: Basic Books (1984).

[25] Lewinsohn PM Graf M. Pleasant events, depression, and locus of control. J Consulting Clin Psychol. (1973) 41:261–4. doi: 10.1037/h0035142 4147832

[26] Timmerman H Pugsley MK. Historical development of pharmacology. Handb Exp Pharmacol. (2006) 171:1–24.

[27] Collier HOJ. The emergence of pharmacology as a disciplined science. Trends Pharmacol Sci. (1989) 10:1–6.2688210

[28] Smith ML Glass GV Miller TI. The Benefits of Psychotherapy, Baltimore. Baltimore, MD: Johns Hopkins University Press (1980).

[29] Barkham M Lambert MJ. Bergin and Garfield's handbook of psychotherapy and behavior change: 50th anniversary edition. 7th ed. Hoboken, NJ: John Wiley & Sons, Inc. (2021).

[30] Rogers CR. On Becoming a Person: A Therapist's View of Psychotherapy. Boston: Houghton Mifflin. (1961).

[31] Hill CE O'Grady KE. The Hill Interaction Matrix: Therapy and Counseling Scales. College Park, MD: University of Maryland (1985).

[32] Truax CB Carkhuff RR. Toward Effective Counseling and Psychotherapy: Training and Practice. Chicago: Aldine Publishing (1967).

[33] Horvath AO Greenberg LS. Development and validation of the working alliance inventory. J Couns Psychol. (1989) 36:223–33. doi: 10.1037/0022-0167.36.2.223

[34] Lambert MJ Bergin AE. Handbook of Psychotherapy and Behavior Change. New York: Wiley (1994).

[35] Frank JD. Persuasion and healing: A comparative study of psychotherapy. Baltimore, MD: Johns Hopkins Univer. Press (1961).

[36] Cochran WG Cox GM. Experimental Designs. Hoboken, NJ: John Wiley & Sons (1957).

[37] Armitage P Berry G. Statistical Methods in Medical Research. Hoboken, NJ: Blackwell Scientific Publications (1987).

[38] Council MR. Streptomycin treatment of pulmonary tuberculosis: A Medical Research Council investigation. BMJ. (1948) 2:769–82. doi: 10.1136/bmj.2.4582.769 PMC2091872 18890300

[39] Hill AB. Principles of medical statistics. Lancet. (1952).

[40] Luborsky L SB Luborsky L. Comparative studies of psychotherapies. Is it true that everyone has one and all must have prizes. Arch Gen Psychiatry. (1975) 32:995–1008. doi: 10.1001/archpsyc.1975.01760260059004 239666

[41] Greenberg G. The book of woe: The DSM and the unmaking of psychiatry. Westminister, MD: Penguin (2014).

[42] Wampold BE. The great psychotherapy debate: Models, methods, and findings. London: Routledge (2013).

[43] Duncan BL Miller SD Wampold BE Hubble MA. The heart and soul of change: Delivering what works in therapy. Washington, D.C.: American Psychological Association (2010).

[44] McWilliams N. Psychoanalytic diagnosis: Understanding personality structure in the clinical process. New York City, NY: Guilford Press (2011).

[45] Barlow DH. The Oxford Handbook of Clinical Psychology. Walton Street, Oxford: Oxford University Press (2010).

[46] Elkin I SM Watkins JT Imber SD Sotsky SM Collins JF Glass DR. General effectiveness of treatments. Arch Gen Psychiatry. (1989) 46:971–82. doi: 10.1001/archpsyc.1989.01810110013002 2684085

[47] Association AP. Evidence-based practice in psychology: APA presidential task force on evidence-based practice. Am Psychol. (2006) 61:271–85. doi: 10.1037/0003-066X.61.4.271 16719673

[48] Kazantzis N L'Abate L Van Widenfelt BM. Handbook of Homework Assignments in Psychotherapy: Research, Practice, and Prevention. New York City, NY: Springer (2005).

[49] Lambert MJ Ogles BM. Bergin and Garfield's Handbook of Psychotherapy and Behavior Change. Hoboken, NJ: John Wiley & Sons (2004).

[50] Carroll KM Rounsaville BJ. A vision of the next generation of behavioral therapies research in the addictions. Addiction. (2007) 102:850–62. doi: 10.1111/j.1360-0443.2007.01798.x PMC2148498 17523974

[51] Chambless DL Hollon SD. Defining empirically supported therapies. J Consulting Clin Psychol. (1998) 66:7–18. doi: 10.1037/0022-006X.66.1.7 9489259

[52] Tolin DF McKay D Forman EM Klonsky ED Thombs BD. Empirically supported treatment: Recommendations for a new model. Clin Psychology: Sci Pract. (2015) 22:317–38. doi: 10.1111/cpsp.12122

[53] Pérez-Álvarez M. Third-generation therapies: Achievements and challenges. Int J Clin Health Psychol. (2012) 12:291–310.

[54] Hayes SC Hofmann SG. Third-wave” cognitive and behavioral therapies and the emergence of a process-based approach to intervention in psychiatry. World Psychiatry. (2021) 20:363–75. doi: 10.1002/wps.20884 PMC8429332 34505370

[55] Hayes SC Luoma JB Bond FW Masuda A Lillis J. Acceptance and commitment therapy: Model, processes and outcomes. Behav Res Ther. (2006) 44:1–25. doi: 10.1016/j.brat.2005.06.006 16300724

[56] Kabat-Zinn J. Mindfulness-based interventions in context: past, present, and future. (2003) American Psychological Association. V10 N2. 144–156. doi: 10.1093/clipsy/bpg016

[57] Lineman MM Bohus M Lynch TR. Dialectical behavior therapy for pervasive emotion dysregulation: Theoretical and practical underpinnings. (2007) Handbook of Emotion Regulation. pp. 581–605.

[58] Arch JJ Craske MG. Acceptance and commitment therapy and cognitive behavioral therapy for anxiety disorders: Different treatments, similar mechanisms? Clin psychology: Sci Pract. (2008) 15:263. doi: 10.1111/j.1468-2850.2008.00137.x

[59] Baer RA. Mindfulness training as a clinical intervention: a conceptual and empirical review. Clin psychology: Sci Pract. (2003) 10:125. doi: 10.1093/clipsy.bpg015

[60] A.P. Association. Diagnostic and Statistical Manual of Mental Disorders. 3rd ed. Washington, D.C.: American Psychiatric Association (1980).

[61] ISO. ISO 9000 series. Geneva, Switzerland: International Organization for Standardization (1987).

[62] Sommerville I. Software Engineering. Boston, MA: Addison-Wesley (1989).

[63] Mullis KB Faloona FA. Specific synthesis of DNA in *vitro* via a polymerase-catalyzed chain reaction. Methods Enzymology. (1987) 155:335–50. doi: 10.1016/0076-6879(87)55023-6 3431465

[64] Odum EP. Basic Ecology. Philadelphia, Pennsylvania: Saunders College Publishing (1983).

[65] Unnevehr LJ Jensen HH. The economic implications of using HACCP as a food safety regulatory standard. Food Policy. (1999) 24:625–35. doi: 10.1016/S0306-9192(99)00074-3

[66] W.H. Organization. WHO Expert Committee on Specifications for Pharmaceutical Preparations: Twentieth Report, Technical Report Series. (1975) Geneva, Switzerland: World Health Organization. 21699061

[67] U.S.F.a.D. Administration. Current Good Manufacturing Practice in Manufacturing, Processing, Packing, or Holding of Drugs Vol. 43. Washington, D.C.: Federal Register (1978).

[68] O.f.E.C.-o.a.D. (OECD). OECD Principles of Good Laboratory Practice (1981). Available online at: https://www.oecd.org/chemicalsafety/testing/good-laboratory-practiceglp.htm. (accessed July 01, 2024)

[69] I.C.f.H.o.T.R.f.P.f.H.U. (ICH). ICH E6: Good Clinical Practice (GCP) (1996). Available online at: https://www.ich.org/page/efficacy-guidelines. (accessed July 01, 2024)

[70] McHugh RK Barlow DH. The dissemination and implementation of evidence-based psychological treatments: A review of current efforts. Am Psychol. (2010) 65:73–84. doi: 10.1037/a0018121 20141263

[71] Waller G. Evidence-based treatment and therapist drift. Behav Res Ther. (2009) 47:119–27. doi: 10.1016/j.brat.2008.10.018 19036354

[72] H.R.a.S. Administration. Health Workforce Shortage Areas U.S. Department of Health and Human Services (2023). Available online at: https://data.hrsa.gov/topics/health-workforce/shortage-areas. (accessed July 01, 2024)

[73] N.A.o.M.I. (NAMI). Mental Health by the Numbers. Arlington, VA: NAMI (2021).

[74] H.R.a.S.A. (HRSA) HRSA. National and Regional Projections of Supply and Demand for Behavioral Health Practitioners: 2013-2025. (2016) Washington, D.C.: U.S. Department of Health and Human Services Health Resources and Services Administration.

[75] P.R. Center. Mobile Fact Sheet. Washington, D.C.: Pew Research Center (2021). Available at: https://www.pewresearch.org/internet/fact-sheet/mobile/.

[76] Statista. Internet usage worldwide – Statistics & Facts. Hamburg, Germany: Statista (2022). Available at: https://www.statista.com/statistics/617136/digital-population-worldwide/.

[77] Kenwright M Marks IM Gega L Mataix-Cols Dr. Computer-aided self-help for phobia/panic via internet at home: A pilot study. Br J Psychiatry. (2004) 184:448–9. doi: 10.1192/bjp.184.5.448 15123511

[78] Proudfoot J Goldberg D Mann A Everitt B Marks I Gray JA. Computerized, interactive, multimedia cognitive-behavioural program for anxiety and depression in general practice. psychol Med. (2003) 33:217–27. doi: 10.1017/S0033291702007225 12622301

[79] King DR EM Tartaglia J Nanda G Tatro NA. Methods for navigating the mobile mental health app landscape for clinical use. Curr Treat Options Psychiatry. (2023) 10:1–15. doi: 10.1007/s40501-023-00288-4 PMC10206563 37360961

[80] Buyya R Vecchiola C Selvi ST. Mastering Cloud Computing. Maryland Heights, MO: Elsevier (2013).

[81] Domingos P. A few useful things to know about machine learning. Commun ACM. (2012) 55:78–87. doi: 10.1145/2347736.2347755

[82] Porter R. The Greatest Benefit to Mankind: A Medical History of Humanity. New York: Harper Collins Publishers (1997).

[83] Mozilla. Shady Mental Health Apps Inch Toward Privacy and Security Improvements, But Many Still Siphon Personal Data (2023). Available online at: https://foundation.mozilla.org/en/blog/shady-mental-health-apps-inch-toward-privacy-and-security-improvements-but-many-still-siphon-personal-data/:~:text=Mozilla's%202022%20mental%20health%20apps,Privacy%20Not%20Included%20warning%20label. (accessed July 01, 2024)

[84] Orcha. What is the Health of Mental Health Apps (2020). Available online at: https://orchahealth.com/health-of-mental-health-apps/. (accessed July 01, 2024)

[85] Huguet A RS McGrath PJ Wozney L Wheaton M Conrod J Rozario S. A systematic review of cognitive behavioral therapy and behavioral activation apps for depression. PloS One. (2016) 11. doi: 10.1371/journal.pone.0154248 PMC4852920 27135410

[86] Neary M BJ Palomares K Mohr DC Powell A Ruzek J Williams LM. A process for reviewing mental health apps: Using the One Mind PsyberGuide Credibility Rating System. Digit Health. (2021) 7. doi: 10.1177/20552076211053690 PMC8558599 34733541

[87] John Torous M MBI. Navigating the Sea of Mental Health Apps Carlat Psychiatry Webinars. Newburyport, MA: Carlat CME Institute (2022). Available at: https://www.thecarlatreport.com/blogs/3-carlat-psychiatry-webinars/post/4201-navigating-the-sea-of-mental-health-apps.

[88] Agarwal S JM Wilcox HC Sharma R Hill R Pantalone E Thrul J. Evaluation of Mental Health Mobile Applications. Rockville (MD: Agency for Healthcare Research and Quality (2022). p. 35675475. 35675475

[89] French LA. The History of Policing America From Militias and Military to the Law Enforcement of Today. Lanham, Maryland: Rowman & Littlefield (2018).

[90] FDA. FDA Guidance Documents and Regulations. Available online at: https://www.fda.gov/. (accessed July 01, 2024)

[91] International Medical Device Regulators Forum (IMDRF) Documents. Washington, D.C.: IMDFR (2024). Available at: https://www.imdrf.org.

[92] Eric Althoff CL. Sandoz and Pear Therapeutics Announce US Launch of reSET-OTM to Help Treat Opioid Use Disorder. East Hanover, NJ: Novartis Media Relations (2019). Available at: https://www.novartis.com/news/media-releases/sandoz-and-pear-therapeutics-announce-us-launch-reset-otm-help-treat-opioid-use-disorder.

[93] Rejoyn. Otsuka and Click Therapeutics Announce the U.S. Food and Drug Administration (FDA) Clearance of Rejoyn™, the First Prescription Digital Therapeutic Authorized for the Adjunctive Treatment of Major Depressive Disorder (MDD) Symptoms (2024). Available online at: https://www.otsuka-us.com/news/rejoyn-fda-authorized. (accessed July 01, 2024)

[94] J.H.a.D.F. n. Continuous Delivery: Reliable Software Releases through Build, Test, and Deployment Automation. Chicago, IL: Martin Fowler (2010).

[95] T.O.o.t.N.C.f.H.I. Technology, What Is HL7® FHIR®. Washington, D.C.: HealthIT.gov. Available at: https://www.healthit.gov/sites/default/files/page/2021-04/What%20Is%20FHIR%20Fact%20Sheet.pdf.

[96] Rodgers MM Pai VM Conroy RS. Recent advances in wearable sensors for health monitoring. IEEE Sensors J. (2014) 15:3119–26. doi: 10.1109/JSEN.2014.2357257

[97] U.S.F.D. Administration. Real-World Evidence (2023). Available online at: https://www.fda.gov/science-research/science-and-research-special-topics/real-world-evidence. (accessed July 01, 2024)

[98] U.S.F.a.D. Administration. Milestones in U.S. Food and Drug Law (2023). Available online at: https://www.fda.gov/about-fda/fda-history/milestones-us-food-and-drug-law. (accessed July 01, 2024)

[99] Price J Drevets W. Neurocircuitry of mood disorders. Neuropsychopharmacology. (2010) 35:192–216. doi: 10.1038/npp.2009.104 19693001 PMC3055427

[100] Emmady P Puderbaugh M. Neuroplasticity. Bethesda, Maryland: StatPearls (2023). Available at: https://www.ncbi.nlm.nih.gov/books/NBK557811/. 32491743

[101] Murrough JW Iacoviello BM Hoch MM Huryk KM Collins KA Cutter GR. A randomized, controlled pilot trial of the Emotional Faces Memory Task: a digital therapeutic for depression. NPJ Digital Med. (2018) 1:21. doi: 10.1038/s41746-018-0025-5 PMC6404739 30854473

